# Effects of Calcium Source on Biochemical Properties of Microbial CaCO_3_ Precipitation

**DOI:** 10.3389/fmicb.2015.01366

**Published:** 2015-12-02

**Authors:** Jing Xu, Yali Du, Zhengwu Jiang, Anming She

**Affiliations:** Key Laboratory of Advanced Civil Engineering Materials, Tongji University, Ministry of EducationShanghai, China

**Keywords:** *S. pasteurii*, ureolytic, bacterial CaCO_3_ precipitation, biochemistry, kinetics

## Abstract

The biochemical properties of CaCO_3_ precipitation induced by *Sporosarcina pasteurii*, an ureolytic type microorganism, were investigated. Effects of calcium source on the precipitation process were examined, since calcium source plays a key role in microbiologically induced mineralization. Regardless of the calcium source type, three distinct stages in the precipitation process were identified by Ca^2+^, NH_4_^+^, pH and cell density monitoring. Compared with stage 1 and 3, stage 2 was considered as the most critical part since biotic CaCO_3_ precipitation occurs during this stage. Kinetics studies showed that the microbial CaCO_3_ precipitation rate for calcium lactate was over twice of that for calcium nitrate, indicating that calcium lactate is more beneficial for the cell activity, which in turn determines urease production and CaCO_3_ precipitation. X-ray diffraction analysis confirmed the CaCO_3_ crystal as calcite, although scanning electron microscopy revealed a difference in crystal size and morphology if calcium source was different. The findings of this paper further suggest a promising application of microbiologically induced CaCO_3_ precipitation in remediation of surface and cracks of porous media, e.g., cement-based composites, particularly by using organic source of calcium lactate.

## Introduction

Since the phenomenon that CaCO_3_ precipitation could be induced by many soil bacteria was revealed (Boquet et al., 1973), research on microbial mineral plugging of porous media has been extensively carried out, especially on the application of bioremediation materials in civil engineering. Compared with traditional repair materials, which include cement grout, mortar, water glass, and epoxy resin, the bio-deposited materials are environmentally friendly and have a better compatibility with civil engineering materials, such as concrete and masonry (De Muynck et al., 2010; Wu et al., 2012). What is more to the point, self-repair can be achieved by using microbial induced deposition (Jonkers et al., 2010).

As a common process in nature, pores or fissures can be filled selectively by microorganisms which generate insoluble compounds inside or outside the cell wall (Ruiz et al., 1988). The mechanism of microbial deposition has been revealed and the feasibility of crack repairing in concrete by *Bacillus pasteurii* immobilized in polyurethane foam was verified (Stocks-Fischer et al., 1999; Bang et al., 2001; Bachmeier et al., 2002). It has been shown that the capillary water adsorption and gas permeability of concrete can be reduced effectively by surface treatment from microbiological mediated deposition, and the composition of bacterial culture medium has a significant impact on the morphology of CaCO_3_ (De Muynck et al., 2008a,b; Van Tittelboom et al., 2010; Achal et al., 2011; Xu et al., 2014). In recent years, bacterial induced deposition in using a non-ureolytic pathway has also been found (Jonkers et al., 2010). By mixing spores of these types of bacteria with fresh state concrete, self-healing of concrete cracks is expected since spores will germinate once cracking occurs and CaCO_3_ precipitation in the open cracks will be triggered by bacterial respiration (Wiktor and Jonkers, 2011; Wang et al., 2014a,b; Xu and Yao, 2014).

For the application of microbiologically induced precipitation in repairing, the primary issue would be the exploration of the biochemical process of the deposition. Since this process involves converting a soluble calcium source into insoluble CaCO_3_, the effect of the type of calcium source is critical. However, prior works concerning the influence of the type of calcium source on bacterial mineralization process are rare (De Muynck et al., 2008a). In this paper, the effect of two different types of calcium sources on the ureolytic microbiologically induced mineralization precipitation was investigated, which aims at providing guidance for further application.

## Experimental Procedures

### Bacterial Strains and Growth Conditions

*Sporosarcina pasteurii* ATCC 11859 was used throughout. Bacteria were cultured in liquid media consisted of 5 g peptone, 3 g meat extract, and 20 g urea per liter of distilled water. Liquid media were sterilized by autoclaving for 20 min at 121°C, then final pH was adjusted to be 9. Cultures were aerobically incubated at 30°C on a water-bath shaker operated at 100 rpm for 24 h. Growth was regularly checked quantitatively under optical microscopy by using a hemocytometer. At the end of the incubation, cultures were washed by repeated centrifugation and resuspension in fresh medium to harvest vegetative cells but to remove dissolved culture constituents and residues. Obtained suspensions were microscopically analyzed to quantify the number of cells present, and the suspension was subsequently kept at 4°C until further use.

### Microbiologically Induced CaCO_3_ Precipitation

Calcium nitrate, instead of calcium chloride, was selected as a representative of inorganic calcium source since chloride ions may be detrimental for the concrete reinforcement. Calcium lactate, which is the most studied organic calcium-containing compound, was selected as a representative of organic calcium source. Groups without bacteria addition were set as control. The composition of liquid medium for each group is shown in **Table 1**, where letters of N, L, B, and C in group labels represent calcium nitrate, calcium lactate, bacteria, and control, respectively. Immediate abiotic CaCO_3_ precipitation usually occurs if the initial concentration of calcium ion is high in an alkaline environment, thus the concentration of calcium source was set to 0.025 mol/L.

**Table 1:** Composition of liquid medium for each group.

Group	Composition
N-B	Peptone 5 g/L, Meat extract 3 g/L, Urea 20 g/L, Calcium nitrate 0.025 mol/L, Bacteria
L-B	Peptone 5 g/L, Meat extract 3 g/L, Urea 20 g/L, Calcium lactate 0.025 mol/L, Bacteria
N-C	Peptone 5 g/L, Meat extract 3 g/L, Urea 20 g/L, Calcium nitrate 0.025 mol/L
L-C	Peptone 5 g/L, Meat extract 3 g/L, Urea 20 g/L, Calcium lactate 0.025 mol/L


For each group, triplicate sets of 250 mL Erlenmeyer flasks containing 80 mL of liquid medium were prepared. Each one was inoculated with live bacteria (10^5^ cells/mL). Bacteria were grown at 30°C on the water-bath shaker operated at 100 rpm for 60 h. At regular intervals, three aliquots from each flask were taken to determine the pH, the amount of soluble Ca^2+^, and the NH_4_^+^ concentration, respectively. For pH measurement, a Mettler Toledo pH probe was used. For soluble Ca^2+^ quantification, the liquid medium was centrifuged and the Ca^2+^ concentration in the supernatant was measured by the EDTA titration method (APHA, 1989). For NH_4_^+^ concentration, an ammonia sensing probe was used. The cell density was measured simultaneously by plating colony-counting method, and the composition of the medium used was the same as the medium for culture growth.

### Characterization of Precipitates

The mineralogy of precipitates was determined by powder X-ray diffraction (XRD) using a Rigaku D/max2550VB3+/PC diffractometer. The morphology of the precipitates was studied by scanning electron microscopy (SEM) using a Hitachi S-2360N. The composition of medium before and after precipitation was dried at 80°C and then analyzed by Fourier transform infrared spectroscopy (FT-IR) using a Bruker Hyperion 2000.

## Results And Discussion

### Biochemical Process of Microbiologically Induced CaCO_3_ Precipitation

**FIGURE 1:**
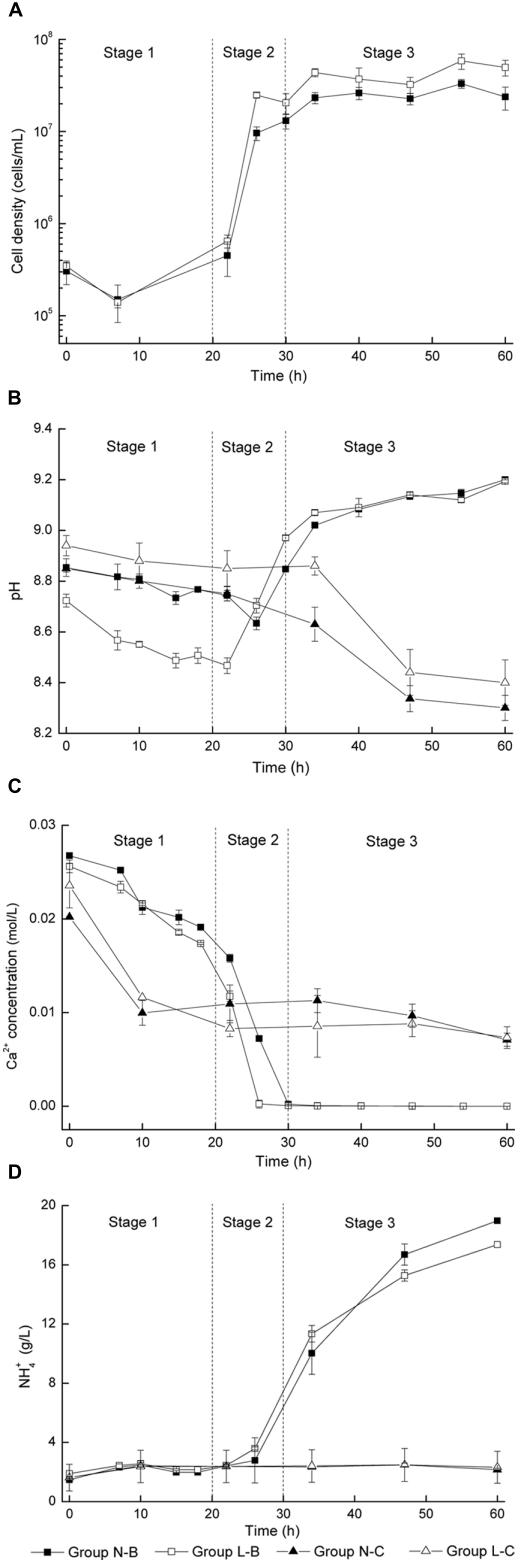
**Microbiologically induced CaCO_3_ precipitation: **(A)** Cell density; **(B)** pH values; **(C)** Soluble Ca^2+^ concentration; **(D)** NH_4_^+^ concentration**.

The growth of bacteria in the mineralized medium is shown in **Figure 1A**. The initial concentration of bacteria in medium was about 10^5^ cells/mL. In the first 22 h of induction period, the cell density showed a little increment of less than 2 × 10^5^ cells/mL. The period of logarithmic growth appeared in 22–26 h and the cell density increased by nearly 100 times. It is observed that bacteria in medium with calcium lactate was more active than in medium with calcium nitrate, for the cell density of group L-B was always two times of that of group N-B. The control groups were not included since no strains were inoculated.

In **Figure 1B**, pH values of viable bacteria groups decreased for about 0.2 in the first 20–25 h, then rose rapidly for about 0.5 in the next 5–10 h, and finally kept steady. For the control groups, pH value variation less than 0.1 was observed in the first 30 h, followed by a remarkable decline around 0.5 in the later period.

**Figure 1C** shows the evolution of soluble Ca^2+^ concentration. The Ca^2+^ concentration of viable bacteria groups decreased rapidly during 20–30 h for about 0.02 mol/L until the free Ca^2+^ was completely converted into precipitates. For the control groups, Ca^2+^ concentration decreased for about 0.01 mol/L in the first 10 h and then kept steady in the following period, indicating that free Ca^2+^ were not combined in the later stage.

In **Figure 1D**, the NH_4_^+^ concentration of viable bacteria groups was nearly 3 g/L in the first 22 h, and then increased significantly to more than 10 g/L. In contrast, the NH_4_^+^ concentration for the control groups always kept constantly at 3 g/L.

The main objective of this paper is to explore the biochemical properties of bacterial induced mineralization, which would be very helpful for its application in structural remediation, particularly in concrete engineering. It was considered that the biochemical process of microbiologically induced CaCO_3_ precipitation consists of three distinct stages with similar pattern, regardless of the type of calcium source (**Figure 1**). Details for each stage is discussed as follows:

In the first stage, pH value and Ca^2+^ concentration dropped gradually while NH_4_^+^ concentration and cell density were almost unchanged. The initial alkalinity of the medium was mainly caused by the decomposition of urea during sterilization. In general, Ca^2+^ ions are apt to precipitate in an alkaline environment, which in turn results in a decrease of pH value. It is interesting to note that a reduction of 0.012 mol/L for Ca^2+^ occurred during this stage for both the groups with viable cells and control groups (**Figure 1B**), which indicates that abiotic precipitation mainly contributes to the deposition of CaCO_3_ in this stage.

At the end of the second stage, the calcium sources were completely converted into CaCO_3_. The biological-chemical mechanism of this process can be expressed as follows

$$CO{\left({NH}_{2}\right)}_{2}+3H_{2}O\overset{Urease}{\to }2{NH}_{4}^{+}+2OH^{-}+CO_{2}↑$$

$$CO_{2}+H_{2}O↔HCO^{-}+H^{+}$$

$$HCO^{-}+H_{2}O↔H^{+}+{CO}_{3}^{2-}$$

$$Cell-{Ca}^{2+}+{CO}_{3}^{2-}\to Cell-CaCO_{3}↓$$

At this stage, the density of bacteria increased by two orders of magnitude, which corresponds to the logarithmic phase of bacterial growth. Urea was decomposed into NH_3_ and CO_2_ by urease, which was produced by bacteria. pH value increased rapidly due to the fact that the large amount of NH_3_ generated by decomposition of urea. An increase of pH value promoted the dissolution of CO_2_ and the hydrolysis reaction of HCO^-^, resulting in an increase of CO_3_^2-^ concentration. In the meantime, positively charged metal ions were bound on bacterial surfaces due to the presence of several negatively charged groups on the cell wall. Such bound metal ions (e.g., Ca^2+^) may subsequently react with anions (e.g., CO_3_^2-^) to form an insoluble salt (e.g., CaCO_3_) (Stocks-Fischer et al., 1999; Bang et al., 2010; De Muynck et al., 2010). Although the duration of stage 2 is short, it should be considered as a critical part in the whole process, and enzymatic hydrolysis by microorganisms is essential in this stage.

For the last stage, depletion of free Ca^2+^ was observed, while the bacteria density almost remained constant around 10^7^ cells/mL, because of the depletion of nutrients and an increased amount of harmful metabolite. NH_3_ and CO_2_ were continuously released due to sustained decomposition of urea. The increase of pH value slowed down because the neutralization effect of CO_2_ with NH_3_.

### Kinetics of Biotic CaCO_3_ Precipitation

**FIGURE 2:**
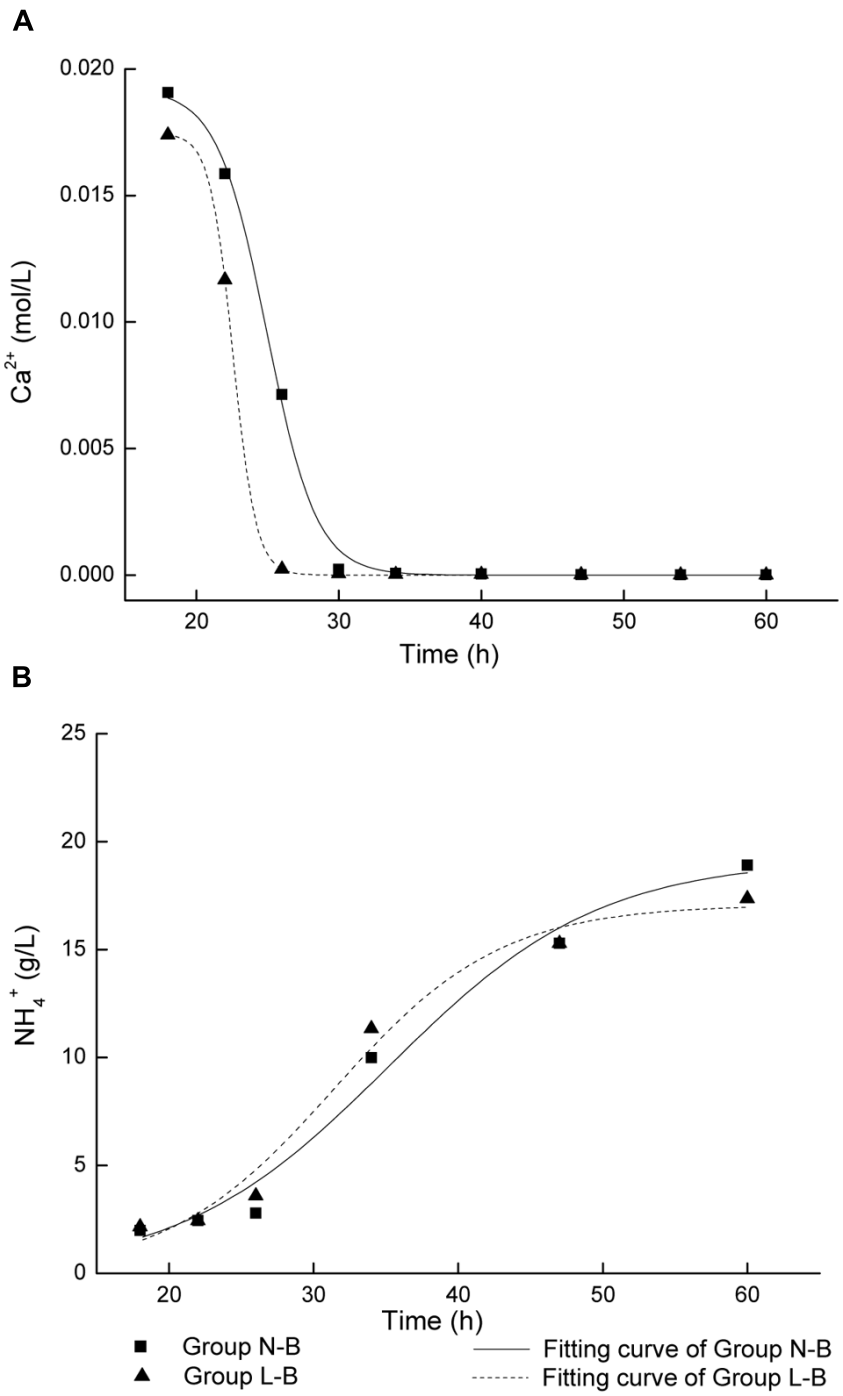
**Fitting of curves for microbiologically induced precipitation with live cells: **(A)** CaCO_3_ precipitation; **(B)** NH_4_^+^ production**.

As presented above, CaCO_3_ precipitation and ammonia production is correlated with cell growth. It could be noted that the initiation of biotic CaCO_3_ precipitation and ammonia production for the group L-B was earlier than that of group N-B (**Figures 1B,D**). In order to further study the effect of calcium source on the biochemical process, kinetics of biotic CaCO_3_ precipitation as well as ammonia production was examined. In **Figure 2**, curves corresponding to biotic CaCO_3_ precipitation (data in stage 1were excluded) were fitted by an exponential logistic equation (Marquardt, 1963)

$$y=\frac{a}{1+e^{-k\left(x-x_{c}\right)}}$$

where *x* is time, *y* is ion concentration, *a* is the variation range of *y*, *x*_c_ is the time when d*y*/d*x* reaches the maximum value, *k* is the rate constant. The *k* values of CaCO_3_ precipitation and ammonia production were calculated from regression analysis, as shown in **Table 2**.

**Table 2:** Rate constants of CaCO_3_ precipitation and ammonium ions production in different calcium source media.

*k* (h^-1^)	Calcium nitrate	Calcium lactate
CaCO_3_ precipitation	0.574	1.239
Ammonia production	0.137	0.174


The rate constants in medium with calcium lactate are larger than that in medium with calcium nitrate. In particular, the *k* value of CaCO_3_ precipitation in calcium lactate is over twice of that in calcium nitrate. It suggests that higher cell activity and faster biotic CaCO_3_ precipitation and ammonia production rate if organic source of calcium lactate was used, which could be due to the bacterial metabolic conversion of calcium lactate according to the following reaction (De Muynck et al., 2010):

$$CaC_{6}H_{10}O_{6}+6O_{2}\to CaCO_{3}↓+5CO_{2}↑+5H_{2}O$$

Calcium lactate is not only the calcium source, but a kind of carbon source that provides additional nutrition for bacteria. Moreover, extra amounts of CO_2_ were released if calcium lactate was decomposed by bacterial metabolism. It was reported that other organic calcium compounds have similar bio-reaction (Jonkers, 2011). In this respect, organic calcium source seems more beneficial for microbial mediated CaCO_3_ precipitation. It is worth to mention that substantial calcium lactate can be obtained from dairy by-products, which can meet the requirements of a potential use of calcium lactate in the field.

### Analysis of Precipitates

**FIGURE 3:**
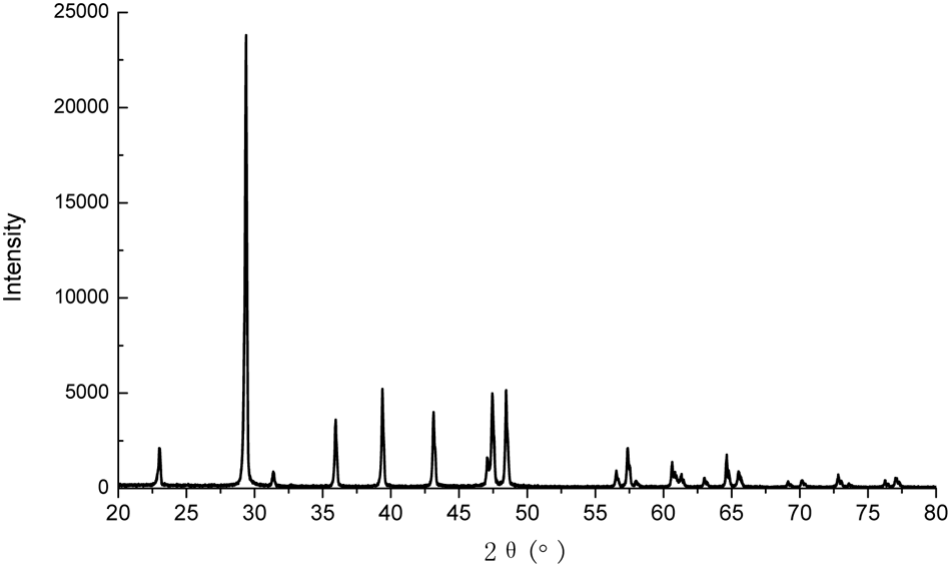
**X-ray diffraction (XRD) pattern of the precipitates**.

Calcite was precipitated in the liquid medium irrespective of the type of calcium source, as confirmed by XRD analysis (**Figure 3**). This is consistent with results of other researchers (Bang et al., 2001; De Muynck et al., 2008a).

**FIGURE 4:**
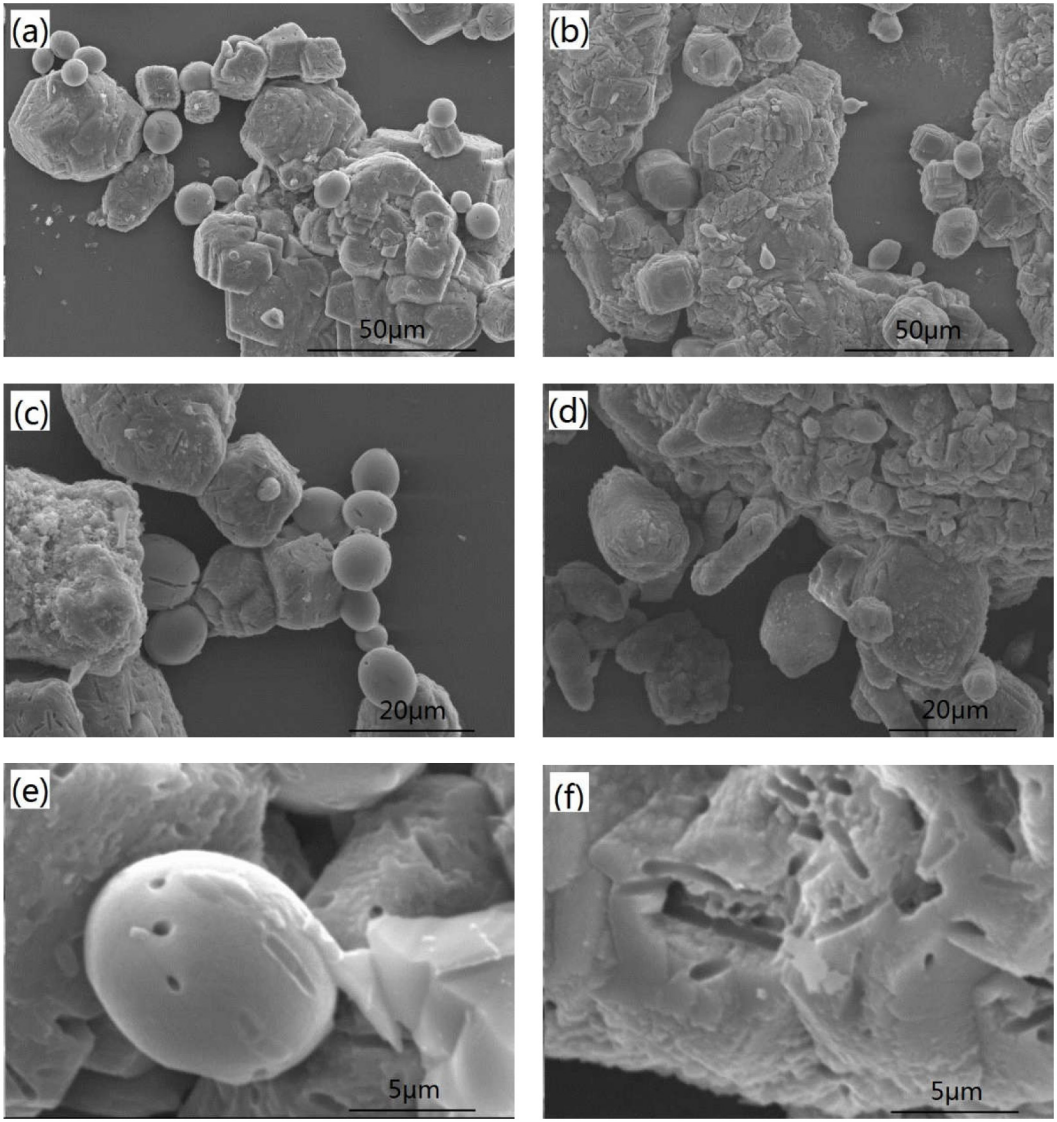
**Scanning electron microscopy (SEM) images of the precipitates from different calcium source: **(a,c,e)** calcium nitrate; **(b,d)** and **(f)** calcium lactate**.

**Figure 4** shows SEM images of calcite precipitates from two kinds of calcium sources. Difference in morphology was observed from calcium nitrate to calcium lactate. The sediments from calcium nitrate are spherical and lamellar particles, with particle size less than 50 μm. In contrast, sediments from calcium lactate are mostly irregular compact lumps or rhombohedral crystals with relatively larger particle size. Imprints of 2–4 μm long and 0.7 μm wide were observed on the surface of crystals. These imprints are likely left by bacteria. Prior findings have already verified that bacterial cells can provide favorable conditions by acting as a nucleus for the formation of crystals (Stocks-Fischer et al., 1999; Bang et al., 2010).

**FIGURE 5:**
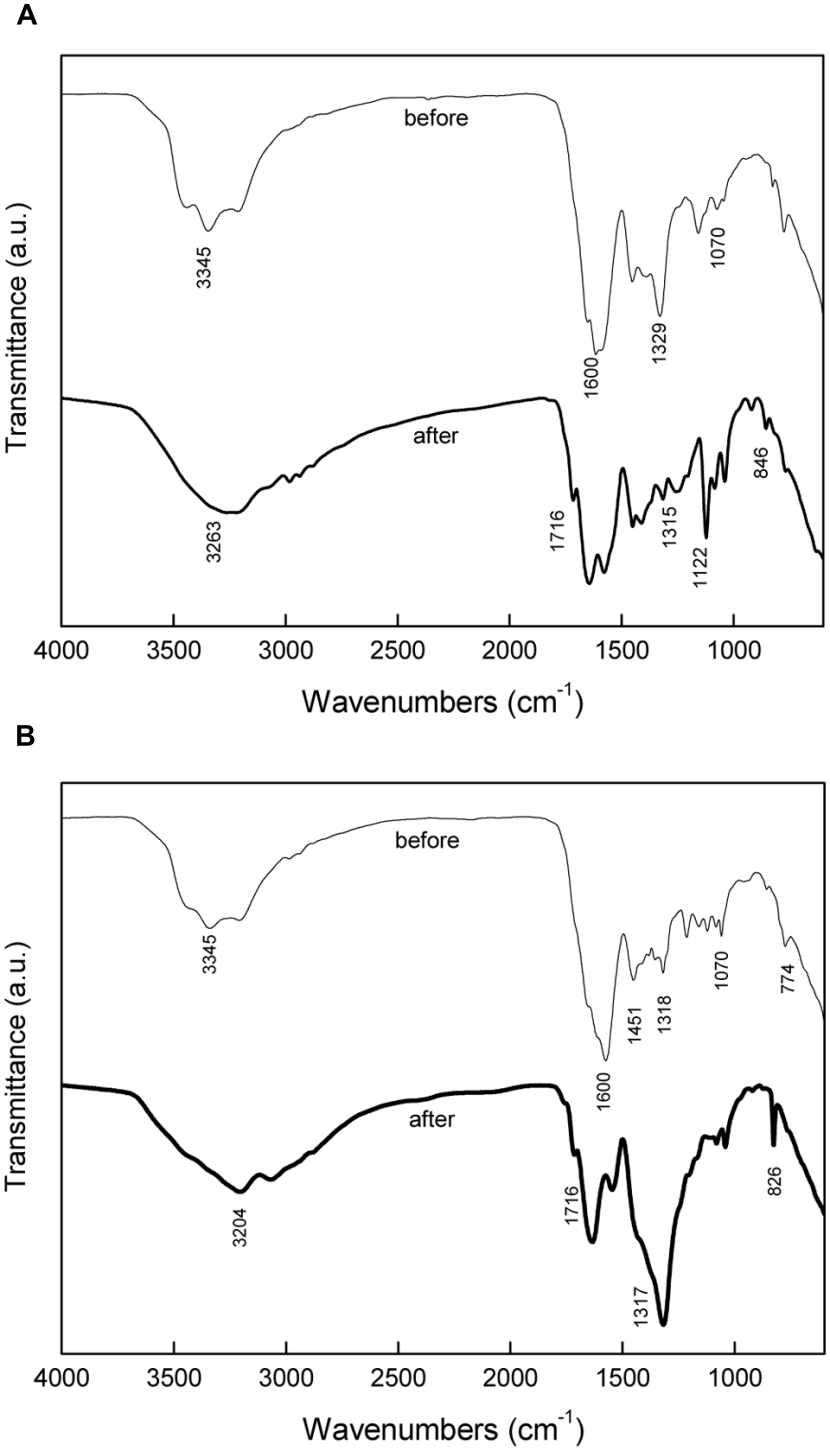
**Fourier transform infrared spectroscopy (FT-IR) analysis of media before and after precipitation: **(A)** inorganic calcium source; **(B)** organic calcium source**.

**Figure 5** shows the FT-IR spectra of medium with different calcium source before and after precipitation. For the medium before precipitation, the presence of urea was indicated by amino signature at 3345 and 1070 cm^-1^ and carboxide signature at 1600 cm^-1^. Calcium nitrate was identified by absorption peak at 1329 cm^-1^. Calcium lactate was identified by absorption bands at 1451, 1318, and 774 cm^-1^. For the medium with calcium nitrate after precipitation, a small broad hump at 3263 cm^-1^ and the absorption band 1315 cm^-1^ correspond to NH_4_^+^, while bands near 1716 cm^-1^, 1122 cm^-1^, and 846 cm^-1^ indicate the presence of CO_3_^2-^. Bands corresponding to NH_4_^+^ and CO_3_^2-^ were also detected for the medium with calcium lactate after precipitation. FT-IR spectra confirms that NH_4_^+^ and CO_3_^2-^ were produced by bacterial metabolism.

Although calcite was obtained irrelevant to the type of calcium source, different crystal size and morphology were observed for calcium nitrate and calcium lactate. This is consistent with other findings showing that the type of calcium source has a significant impact on the crystallization process (De Muynck et al., 2008a). The crystal growth can be inhibited or altered by the adsorption of organic or inorganic matters to specify crystallographic planes of the growing crystal (Rodriguez-Navarro et al., 2007). On the other hand, differences in crystal morphology could be due to the level of the actual urease activity, which correlates with the cell activity. It further confirms the kinetics results demonstrating that cell activity differs from inorganic calcium nitrate to organic calcium lactate. The influence of conditions other than calcium source type, such as pH, temperature, composition and concentration of nutrients on the morphology of precipitates, are worthy of further research. The influence of morphology and crystal size of on the properties of CaCO_3_ precipitates also needs assessment in civil engineering remediation application.

## Conclusion

The biochemical investigations revealed that the microbiologically induced CaCO_3_ precipitation consists of three distinct stages and is independent of calcium source. The rate of precipitation for calcium lactate was over twice of that for calcium nitrate, indicating that organic source of calcium lactate may be more beneficial for the cell activity, which directly related to urease production and CaCO_3_ deposition. The CaCO_3_ crystal were identified as calcite, although the morphology varied if the type of calcium source was different. It also confirmed that bacterial cells acted as nucleation sites for crystal formation and growth.

## Author Contributions

JX, corresponding author and the main contributor of the paper; YD, contribute to the main part of experimental work of the paper; ZJ, provide some ideas and part of funding to the work; AS, provide some ideas and part of testing of the paper.

## Conflict of Interest Statement

The authors declare that the research was conducted in the absence of any commercial or financial relationships that could be construed as a potential conflict of interest.
